# Inhibitory Effects and Transcriptomic Insights of L-Arginine Against *Diaporthe nobilis*

**DOI:** 10.3390/jof12100783

**Published:** 2026-10-19

**Authors:** Shuang Zhou, Xuesong Wang, Ziye Wang, Lifeng Duan, Jianhua Feng, Dong-Hui Yan

**Affiliations:** 1 Key Laboratory of Ecology and Nature Conservation Institute, Chinese Academy of Forestry, Beijing 100091, China; zhoushuang@caf.ac.cn (S.Z.); wangzy@caf.ac.cn (Z.W.); 2 Liaoning Forestry Development Service Center, Shenyang 110031, China; qiulvhua@163.com; 3 Beijing Coway BioWorks Biotech Co., Ltd., Beijing 100084, China; dlf@ittc.com.cn; 4 Liangjiayoufang Central Forest Farm of the Sanggan River Poplar High-Yield Forest Experimental Bureau, Youyu County, Shuozhou 037200, China; fjhllq2002@163.com

**Keywords:** L-Arg, *Diaporthe nobilis*, postharvest storage, chestnuts, transcriptomics

## Abstract

*Diaporthe nobilis* causes postharvest decay in Chinese chestnuts (*Castanea mollissima* Blume). This study evaluated the effects of exogenous L-arginine (L-Arg) on fungal growth, fruit decay, and transcriptional responses using a single *D. nobilis* isolate. In a preliminary in vitro screen of nine amino acids at 30 mM, L-Arg showed the strongest inhibition of mycelial growth. L-Arg inhibited mycelial growth in a concentration-dependent manner in vitro. In the single fruit assay, the 2.5–20 mM groups had lower observed disease incidence and disease indices than the water control, whereas the 30 mM group had values close to the control. Transcriptomic analysis of mycelia grown on potato dextrose agar (PDA) showed lower transcript levels for several genes annotated as encoding membrane transporters, cell wall-degrading enzymes (CWDEs), and proteins involved in secondary metabolism under L-Arg treatment. These findings describe transcriptional responses under in vitro culture conditions. Without functional or biochemical validation, they do not establish reduced CWDE activity or suppression of virulence during host infection. The contrasting responses of fungal growth and fruit decay indicate that stronger inhibition in vitro does not necessarily translate into better disease control. Further studies across isolates, together with measurements during infection and functional validation, are needed to assess the mechanisms and practical value of L-Arg for postharvest chestnut protection.

## 1. Introduction

*Diaporthe* species are widely distributed and serve as fungal pathogens of many plants and even mammals besides being non-pathogenic endophytes (biotrophic fungi), saprobes [1,2]. Plant pathogenic *Diaporthe* spp. can cause wood cankers, wilts, dieback, and fruit rot in various economically valuable plant hosts, particularly affecting economically important crops such as soybeans, sunflowers, grapes, citrus, chestnuts, and ornamental trees [3]. Rot diseases caused by *Diaporthe* species are prevalent and highly destructive in postharvest fruits including chestnut fruit [4,5,6,7,8]. Chestnut rot diseases pose a severe threat to the economic benefits of chestnuts and outbreak typically peaks during the postharvest storage period. Although a diverse range of pathogens in chestnut fruit rots were identified such as *Diaporthe*, *Alternaria*, *Botryosphearia*, and *Colletotrichum* genera, the *Diaporthe* fungi always take dominant abundance among these genera [9]. Furthermore, *Diaporthe nobilis* has recently been identified as a major causal pathogen for fruit rots of *Castanea mollissima* var. Yanshanzaofen, which is the most economically profitable chestnut variety in the Yanshan region of northern China and recorded as a very susceptible variety to rot diseases, with a cultivation area exceeding 66,667 hectares and an export volume accounting for more than 50% of the total chestnut exports [10]. Currently, chemical pesticides are commonly used to control *Diaporthe*-induced diseases [11,12]. However, the excessive use of chemical pesticides has led to serious issues such as food contamination, environmental pollution, and phytotoxicity [13]. Consequently, as consumer concern about food safety and environmental issues continues to rise, ecologically friendly postharvest preservation technologies have developed rapidly, including plant essential oils and natural antifungal compounds such as curcumin [14,15].

L-Arg is predominantly present as a structural amino acid in proteins, and it plays an essential role in protein synthesis and degradation. It is widely distributed in most prokaryotic and eukaryotic organisms [16,17]. In plants, L-Arg is involved in growth, development, and stress tolerance through its conversion to putrescine via ornithine, which is critical for root elongation and development. A reduction in putrescine inhibits root elongation, whereas exogenous putrescine (0.01–1 mM) promotes root growth and system development [18,19,20,21,22,23,24,25]. L-Arg is also hydrolyzed by L-Arginase to produce urea, which is integrated into the nitrogen recycling pathway, supporting plant development and responses to biotic and abiotic stresses [26].

In fungi, L-Arg supplementation enhances the production of perylenequinone in the endophytic fungus *Shiraia* sp. Slf14(w) by upregulating glycolysis-related genes and activating the nitric oxide–cyclic guanosine monophosphate–protein kinase G (NO-cGMP-PKG) signaling pathway [27]. Similarly, L-Arg treatment in postharvest jujube fruit induces resistance to *Alternaria alternata* by balancing reactive oxygen species (ROS) metabolism and enhancing pathogenesis-related protein activity [28]. Additionally, L-Arg regulates microcycle conidiation in *Metarhizium acridum* by modulating nitric oxide (NO) levels, with disruption of the arginase gene (MaAGA) leading to reduced NO and altered sporulation patterns [29].

This study examined the in vitro growth and transcriptional responses of *D. nobilis* to exogenous L-Arg and recorded decay in inoculated chestnuts after L-Arg soaking. We aimed to describe concentration-related patterns and identify candidate pathways for further investigation; the fruit outcomes were assessed descriptively.

## 2. Materials and Methods

### 2.1. Fruit, Strains, and Chemicals

The chestnut variety used in this study is Yanshanzaofeng harvested from Qianxi County in Tangshan City, Hebei Province, which is the core distribution area of Yanshanzaofeng. Healthy chestnuts were stored in a 4 °C refrigerator for preservation.

The isolate BLBF-1 used in this study was obtained from naturally diseased chestnut fruits collected in the field. In a previous study, it was assigned to *Diaporthe nobilis* on the basis of ITS phylogenetic analysis considered together with available colony and conidial morphology; its pathogenicity on chestnut fruits was also reported [9]. The isolate was cultured on PDA medium for subsequent experiments. No GenBank accession is currently available for BLBF-1, and TEF1 and TUB2 have not been sequenced for this isolate.

L-Arg (free base, purity: 99%) was purchased from Shanghai Macklin Biochemical Technology Co., Ltd., Shanghai, China.

### 2.2. Fruit Decay Assay with L-Arg Treatment

Commercial sodium hypochlorite solution (analytical grade; Fuchen (Tianjin) Chemical Reagent Co., Ltd., Tianjin, China; labelled available chlorine ≥10.0%) was diluted to 2% (*v*/*v*) with water. On the basis of the labelled minimum, the 2% (*v*/*v*) working dilution would contain at least 0.2% available chlorine; its concentration was not measured directly. The chestnuts were washed, immersed in this solution for 3 min, and rinsed with distilled water (dH_2_O). The L-Arg soaking solutions were prepared with sterile dH_2_O, passed through a 0.22 μm membrane filter, and used without pH adjustment. Each treatment group of 24 chestnuts, including the sterile-water control, was soaked together in 2 L of its assigned solution for 24 h; all fruits were fully submerged. This corresponds to approximately 83 mL of solution per fruit as a group-volume ratio. The solutions were not renewed during this period. The L-Arg concentrations were 2.5, 5, 10, 20, and 30 mM. In a subsequent check of freshly prepared solutions at approximately 25 °C, the pH values at 0, 2.5, 5, 10, 20, and 30 mM L-Arg were 5.55, 9.90, 10.15, 10.30, 10.45, and 10.55, respectively. Each value is the mean of three readings from one solution preparation. Using a 5 mm sterile punch, holes were made in the chestnut shells, ensuring that the inner seed coat detached without damaging the seed. Sterile tweezers were used to transfer *D. nobilis* agar plugs, with the mycelium facing the seed, into the holes for inoculation. The fruits were then placed into sterile 150 mm Petri dishes, sealed with cling film, and stored in a 25 °C constant-temperature incubator. After 15 days of dark incubation, the chestnuts were opened, and the disease condition of each group was examined. The fruit assay was conducted once, with 24 chestnuts per treatment group. All the required tools were sterilized in an autoclave at 121 °C for 20 min. All operations were performed in a laminar flow hood.

The disease severity of chestnut rot (disease grade) was classified based on the observed diseased area on the maximum longitudinal section of the chestnut at the inoculation site, using a 5-level scale: Level 0: The seed is healthy with no disease. Level 1: Disease at the inoculation site, with minimal spread; the lesion diameter ≤ 5 mm. Level 2: Lesion has spread, and the diseased area is ≤25%. Level 3: Lesion has spread, and the diseased area is >25% but ≤50%. Level 4: Lesion has spread, and the diseased area is >50%.

The disease index is calculated as:

$$\mathrm{Disease Index}=\frac{\Sigma (\mathrm{Disease Grade}\times \mathrm{Number of Fruits})}{\mathrm{Total Number of Fruits}\times \mathrm{Highest Grade Value}}\times 100$$

### 2.3. Exogenous L-Arg Induced Mycelial Growth Inhibition Assay for D. nobilis

A preliminary amino acid screen was conducted using PDA supplemented with alanine (Ala), glycine (Gly), glutamic acid (Glu), aspartic acid (Asp), lysine (Lys), arginine (Arg), leucine (Leu), valine (Val), or γ-aminobutyric acid (GABA), each at a final concentration of 30 mM. Unsupplemented PDA served as the control. Colony growth was recorded daily under the same in vitro culture conditions (Figure S1). For the L-Arg dose–response assay, PDA was autoclaved at 121 °C for 20 min and cooled to 50–60 °C. Stock solutions of L-Arg were filter-sterilized using a 0.22 μm membrane filter (to avoid high-temperature Maillard reactions with sugars) and amended into the autoclaved PDA medium to achieve final concentrations of 2.5 mM, 5 mM, 10 mM, 20 mM, and 30 mM. In a subsequent check, the corresponding potato–dextrose liquid medium without agar was measured at approximately 25 °C. At 0, 2.5, 5, 10, 20, and 30 mM L-Arg, the mean pH values were 7.20, 8.85, 9.15, 9.40, 9.65, and 9.80, respectively; each mean was based on three readings from one preparation. These values do not represent the pH of the final solidified L-Arg-amended PDA, which was not measured. An equal volume of sterile water was used as a control sample. A 5 mm mycelial disc of *D. nobilis* (the disc was obtained by punching from a PDA culture after 7 days of incubation) was cultured on the PDA medium containing L-Arg. The colony diameter was measured daily using a ruler. For each treatment, independent Petri dishes (*n* = 5) were evaluated, and three colony diameters were measured in different directions on each plate. The total colony diameter increase (mm) was evaluated over the 5-day active incubation period from Day 1 to Day 6 (D_Day 6_ − D_Day 1_). The daily growth rate (mm/d) was calculated as the mean incremental diameter extension across consecutive 24 h intervals (Days 1–6):

$$\mathrm{Daily Growth Rate}=\frac{1}{5}\sum_{t=2}^{6}\left(D_{t}-D_{t-1}\right)$$

The antifungal activity was determined by radial mycelial growth bioassay, and the inhibition rate was calculated as follows:

$$\mathrm{Inhibition rate}=\frac{C-T}{C-5}\times 100\%$$

C = the colony diameter of *D. nobilis* on the control plate;

T = the colony diameter of *D. nobilis* on the treatment plate;

5 = the initial size of the *D. nobilis* mycelial block

### 2.4. Transcriptome Analysis of D. nobilis Treated with L-Arg

To investigate the inhibitory mechanism of L-Arg on *D. nobilis*, three treatment groups were prepared: untreated control (CK), low-concentration L-Arg (2.5 mM), and high-concentration L-Arg (30 mM). Sterilized glass paper was placed on PDA plates supplemented with the filter-sterilized L-Arg concentrations (2.5 mM and 30 mM), and 5 mm mycelial discs of *D. nobilis* were inoculated and incubated in the dark at 25 °C for 7 days. For each treatment, mycelia from five Petri dishes were pooled as one sample, with three biological replicates (*n* = 3).

RNA extraction, library preparation, and high-throughput sequencing were conducted by Kangsheng Xuanyuan Biotechnology (Wuhan) Co., Ltd. (Wuhan, China). Total RNA purity and integrity were evaluated using a NanoDrop 2000 spectrophotometer (Thermo Fisher Scientific, Wilmington, DE, USA) and an Agilent 2100 Bioanalyzer (Agilent Technologies, Santa Clara, CA, USA). Poly(A) mRNA was enriched using Oligo(dT) beads (Thermo Fisher Scientific, Waltham, MA, USA), randomly fragmented, and converted into cDNA libraries. Libraries were quantified using Qubit 3.0 (Thermo Fisher Scientific, Waltham, MA, USA) and qPCR (>2 nM) before paired-end 150 bp (PE150) sequencing on an Illumina platform (Illumina, San Diego, CA, USA).

Raw sequencing reads were filtered to remove adapters and low-quality reads, yielding 90.11 Gb of clean data (Q30 ≥ 94.33%). Because a genome sequence had not been generated for the *D. nobilis* isolate used in this study, de novo assembly was used to construct the reference for expression analysis. Trinity [30] produced 60,895 transcripts, which were represented by the longest sequence within each Trinity gene group, giving 44,050 unigenes with an N50 of 2232 bp. We verified this representative-sequence selection by comparing the transcript and unigene FASTA files. All 44,050 unigene sequences were distinct in an exact sequence comparison. Here, a unigene denotes an assembled representative sequence, not a validated genomic locus; distinct representatives may still correspond to isoforms, allelic variants, or fragments of the same gene. Expression was quantified against the representative unigene sequences using RSEM (v1.2.19) [31].

Assembly completeness was evaluated on the final 44,050 representative unigene sequences with BUSCO v5.8.0 in transcriptome mode using the sordariomycetes_odb10 lineage dataset (3817 ortholog groups).

Functional annotation was performed using DIAMOND (v2.0.4; E-value ≤ 10^−5^) against the NCBI non-redundant protein (NR), Swiss-Prot, COG, KOG, eggNOG, and KEGG databases [32,33,34,35,36,37]. Additional annotations were obtained using HMMER (v3.1b2) against Pfam and InterProScan (v5.34-73.0) for Gene Ontology (GO) assignments [38,39]. InterProScan was used for protein-function classification [40].

Clean reads were mapped to the assembled unigenes using Bowtie2 (v2.3.5.1) [41], and gene expression abundances were quantified as FPKM (Fragments Per Kilobase of transcript per Million mapped reads) values using RSEM [31]. For differential expression analysis, raw read counts were normalized and analyzed using DESeq2 (v1.30.1) [42]. Differentially expressed unigenes, referred to below as DEGs, were identified using the threshold of |log2 (Fold Change)| ≥ 1 (representing a ≥ 2-fold increase or decrease in relative expression) and a False Discovery Rate (FDR) < 0.01. Here, FPKM values represent normalized transcript expression levels across individual samples, whereas log2 (Fold Change) reflects the relative magnitude of expression change between treatment and control groups.

GO and KEGG over-representation analyses were performed using the hypergeometric test in clusterProfiler. The foreground comprised the combined upregulated and downregulated DEGs for each comparison. The background was the annotated unigene reference used in the original enrichment analysis, rather than the DEG list alone. The effective background sizes were 7814 for biological process (BP), 8410 for cellular component (CC), 12,927 for molecular function (MF), and 6636 for KEGG. These sizes were checked against the supplied annotation tables and reproduced the reported hypergeometric *p*-values. This background was based on annotated assembly units and was not restricted by an additional expression filter during the revision. Enrichment computations used clusterProfiler as described by Yu et al. [43].

GeneRatio was defined as *k*/*n*, where *k* is the number of DEGs assigned to a term and *n* is the number of DEGs represented in the corresponding GO or KEGG analysis. For the 2.5 mM versus CK, 30 mM versus CK, and 30 mM versus 2.5 mM comparisons, respectively, *n* was 239, 694, and 752 for BP; 372, 1060, and 1059 for CC; 638, 1706, and 1732 for MF; and 347, 912, and 913 for KEGG. BgRatio was defined as K/N, where K is the number of background units assigned to the term and N is the corresponding effective background size. Each identifier was counted once per term. For each comparison, Benjamini–Hochberg correction was applied across all GO terms listed in the original results, pooling BP, CC, and MF, and separately across all KEGG pathways listed in the original results. Terms with adjusted *p* < 0.05 were considered significantly enriched. The enrichment plots display up to 10 terms per GO ontology and up to 20 KEGG pathways, respectively, ranked by adjusted *p*-value.

Reverse transcription was performed using the TRUEscript RT MasterMix kit (Aibosen, Beijing, China). Target gene primers were designed using Primer Premier 5 [44], with the actin gene selected and verified as a stable internal reference control. The expression stability of the internal reference gene (actin) across all treatment conditions (CK, 2.5 mM, and 30 mM L-Arg) was confirmed, showing negligible variation among group means (Cq = 22.19 ± 0.62 for CK, 22.23 ± 0.62 for 2.5 mM, and 22.27 ± 0.94 for 30 mM L-Arg; overall Cq = 22.23 ± 0.71). RT-qPCR was conducted using 2× SYBR Green qPCR Mix (Tiangen Biotech Co., Ltd., Beijing, China) on a CFX96 Real-Time PCR Detection System (Bio-Rad Laboratories, Hercules, CA, USA). The reaction mixture (25 µL) comprised 12.5 µL 2× SYBR Green qPCR Mix, 0.5 µL of each primer (10 µM), 1 µL cDNA template, and 10.5 µL ddH_2_O. Thermal cycling conditions were: 95 °C for 2 min, followed by 40 cycles of 95 °C for 15 s and 60 °C for 30 s. Amplicon specificity was verified via melt-curve analysis (a single sharp peak per product) and agarose gel electrophoresis. Three independent biological replicates (each with three technical replicates) were analyzed for each treatment (*n* = 3). Relative gene expression was calculated using the 2^−ΔΔCt^ method. Primer sequences are provided in Supplementary Table S1.

Cycle-by-cycle fluorescence data from the eight RT-qPCR runs were analyzed using RDML-Python LinRegPCR (version 1.7.2). Mean amplification-curve efficiencies and exponential-phase fit R^2^ values for each assay are given in Supplementary Table S1.

### 2.5. Statistical Analysis

All in vitro assays, including mycelial growth bioassays (*n* = 5 independent biological replicates per treatment) and RT-qPCR quantitative assays (*n* = 3 biological replicates per treatment), were conducted with independent biological replicates, and data are presented as the mean ± standard deviation (SD). Differences among multiple treatment groups for in vitro assays were evaluated using one-way analysis of variance (ANOVA) followed by Dunnett’s post hoc test (for comparing treatment groups against the control CK) or Tukey’s post hoc test (for multiple pairwise comparisons). For the postharvest chestnut decay evaluation (*n* = 24 individual fruits per treatment group), disease incidence, the grade distribution, and the disease index are summarized descriptively from the 24 fruits in each treatment. Each disease index is a single pooled value; no inferential test was applied to the fruit data. Statistical analyses and plotting were performed using GraphPad Prism (version 8.0) or R software (v4.5.2) [45,46]. Statistical significance was defined at *p* < 0.05 (* *p* < 0.05, ** *p* < 0.01, *** *p* < 0.001, ns: not significant).

## 3. Results

### 3.1. Inhibitory Effect of L-Arg on D. nobilis In Vitro

Treatment of *D. nobilis* with different types of amino acids revealed that Ala, Gly, Glu, Asp, Lys, and L-Arg inhibited mycelial growth, whereas Leu, Val, and GABA promoted growth, with L-Arg exhibiting the most significant inhibitory effect (Figure S1). Exogenous L-Arg affects the growth of *D. nobilis*. Treatment with different concentrations of L-Arg shows that the inhibitory effect on *D. nobilis* colony growth becomes more pronounced as the concentration increases, with noticeable inhibition at 2.5 mM, 5.0 mM, 10 mM, 20 mM, and 30 mM (Figure 1A–F). Over the 5-day active incubation period (from Day 1 to Day 6), higher concentrations of L-Arg led to stronger inhibition of *D. nobilis* colony extension (Figure 1G). Specifically, the net colony diameter increase (*D*_Day 6_ − *D*_Day 1_) was 66.13 mm at 2.5 mM, while at 30 mM, it increased by 24.34 mm (Figure 1G). In terms of mean daily growth rate calculated across consecutive 24 h intervals (Days 1–6), the rate was 13.23 mm/d at 2.5 mM, whereas at 30 mM, it was reduced to 4.87 mm/d (Figure 1H). Compared with the control group (0 mM), the inhibitory effect of 30 mM L-Arg on colony growth reached 66.9%, while the inhibition at 2.5 mM was 9.65% (Figure 1I). At high concentrations of L-Arg, not only is *D. nobilis* growth inhibited, but the colony color also deepens (Figure 1F).

### 3.2. Chestnut Decay Observed After L-Arg Treatment

According to the disease grading criteria in Section 2.2, the severity of chestnut fruit disease is classified into five levels: grade 0 (Figure 2A,B), grade 1 (Figure 2C,D), grade 2 (Figure 2E,F), grade 3 (Figure 2G,H), and grade 4 (Figure 2I,J). Grade 0 indicates completely asymptomatic and is considered healthy, while grade 4 represents severe decay.

In the CK group, disease incidence reached 100% with the highest disease index of 91.67%, with the majority of fruits showing severe grade 3 or 4 decay (Figure 2K,L). At 2.5–20 mM, L-Arg treatment was associated with less observed rot than the control. Specifically, disease incidence was 50% at 2.5 mM, 33.3% at 5.0 and 10 mM, and 16.7% at 20 mM; most fruits in these groups were graded 0 or 1 (Figure 2K). The disease indices were 39.58%, 20.83%, 22.92%, and 10.42% for the 2.5, 5.0, 10, and 20 mM treatment groups, respectively (Figure 2L). At 30 mM L-Arg, disease incidence was 100% and the disease index was 89.58%, with grade 3 or 4 lesions observed (Figure 2K,L). The observed disease indices showed a non-monotonic concentration response: the 30 mM group did not show the lower disease index observed at 2.5–20 mM. These pooled fruit outcomes do not establish the cause of the symptom rebound.

### 3.3. Transcriptomic Responses of PDA-Grown D. nobilis to L-Arg

#### 3.3.1. Differential Expression Gene (DEG) Data Analysis

To understand the effects of different L-Arg concentrations on *D. nobilis*, transcriptome analysis was performed on three sample groups. As shown in Table S2, the sequencing data across all samples were relatively balanced, ranging from 9.27 Gb to 10.58 Gb, indicating sufficient data volume. The GC content of each sample was approximately 55%, suggesting stable nucleotide composition without AT or GC bias, and overall high sequencing quality. The Q30 scores also supported adequate read quality. The correlation heatmap and principal component analysis (PCA) (Figures S2 and S3) showed that the CK and 30 mM replicates clustered relatively closely, whereas the 2.5 mM samples were more dispersed. Some between-treatment correlations exceeded the correlation between two 2.5 mM replicates. Given the dispersion of the 2.5 mM replicates, particularly A2.5-1 in the PCA, the low-dose differential-expression and enrichment results should be considered exploratory.

BUSCO identified 3730 complete ortholog groups (97.7%), including 3689 single-copy (96.6%) and 41 duplicated (1.1%) groups; 32 (0.8%) were fragmented and 55 (1.4%) were missing (Supplementary Table S6).

Using |log2 (Fold Change)| ≥ 1 and FDR < 0.01, the gene expression differences among the three groups were compared. As shown in Table S3 and Figure 3, in the 2.5 mM treatment group, a total of 1112 differentially expressed genes (DEGs) were identified (Figure 3A), with 573 genes upregulated (Figure 3B) and 539 genes downregulated (Figure 3C). In the 30 mM treatment group, 3296 DEGs were found (Figure 3A), with 1851 genes upregulated (Figure 3B) and 1445 genes downregulated (Figure 3C). When comparing the 2.5 mM and 30 mM treatment groups, 3370 DEGs were identified (Figure 3A), with 1934 genes upregulated (Figure 3B) and 1436 genes downregulated (Figure 3C). Among the 253 DEGs shared by all three comparisons, 78 were upregulated in every comparison and 98 were downregulated in every comparison (Figure 3). The remaining 77 showed mixed directions: 35 were downregulated at 2.5 mM relative to CK and upregulated at 30 mM relative to 2.5 mM, whereas 42 showed the opposite pattern. For these two groups, the 30 mM versus CK comparison comprised 34 upregulated and 1 downregulated DEG, and 7 upregulated and 35 downregulated DEGs, respectively. Thus, the 253 common DEGs include 176 with consistent directions and 77 with non-monotonic responses across the three concentrations. The full identifiers, log2 FC values, FDR values, and direction patterns are provided in Table S5. These comparisons indicate a broader transcriptional response at 30 mM than at 2.5 mM.

#### 3.3.2. GO Enrichment Analysis

GO enrichment differed among the three comparisons (Figure 4). In the 2.5 mM L-Arg versus CK comparison, only two terms met the adjusted *p* < 0.05 threshold: integral component of membrane (CC; 276 DEGs; GeneRatio = 0.742; adjusted *p* = 2.56 × 10^−31^) and transmembrane transporter activity (MF; 85 DEGs; GeneRatio = 0.133; adjusted *p* = 1.34 × 10^−12^). No BP terms were significantly enriched in this comparison. In the 30 mM L-Arg versus CK comparison, 23 terms were significant: seven BP, two CC, and 14 MF terms. These included amino acid transport, carbohydrate metabolic process, and transmembrane transport in BP; integral component of membrane in CC; and transmembrane transporter activity, monooxygenase activity, and iron ion binding in MF. The comparison between 30 mM and 2.5 mM L-Arg yielded 18 significant terms: five BP, two CC, and 11 MF terms. BP terms included transcription, DNA-templated, amino acid transport, methylation, carbohydrate metabolic process, and carbohydrate transport. Membrane components and transporter activity were enriched in all three comparisons, whereas a wider range of metabolic and transport functions was represented in the comparisons involving 30 mM L-Arg. These results identify functions associated with differential expression; they do not by themselves establish changes in membrane integrity or biochemical activity.

#### 3.3.3. KEGG Enrichment Analysis

KEGG analysis identified 6, 19, and 28 significantly enriched pathways in the 2.5 mM L-Arg versus CK, 30 mM L-Arg versus CK, and 30 mM versus 2.5 mM comparisons, respectively (adjusted *p* < 0.05; Figure 5). Phenylalanine metabolism had the lowest adjusted *p*-value in both comparisons with CK. At 2.5 mM, the other significant pathways were arginine and proline metabolism, aflatoxin biosynthesis, glycine, serine and threonine metabolism, beta-alanine metabolism, and propanoate metabolism. The 30 mM versus CK comparison also showed enrichment of tryptophan metabolism, starch and sucrose metabolism, and ABC transporters. In the comparison between the two L-Arg concentrations, glycine, serine and threonine metabolism was the most significantly enriched pathway, followed by phenylalanine metabolism and fatty acid biosynthesis. Arginine and proline metabolism was enriched in all three comparisons. These findings link the transcriptional response to amino acid metabolism, but enrichment of combined upregulated and downregulated genes does not establish pathway activation or the direction of metabolic flux. Annotation to aflatoxin biosynthesis also does not establish aflatoxin production by this isolate.

#### 3.3.4. Expression Profiles of Annotated Candidate Genes

Representative DEGs were selected from annotations related to transport, secondary metabolism, degradation, and organic substance metabolism (Figure 6). These categories summarize putative functions rather than experimentally verified roles in virulence. Heatmap values were calculated as log_2_(mean FPKM + 1), using the arithmetic mean of the three biological replicates in each treatment. The same numerical scale, from 0 to 11, was used in all panels without row-wise scaling. Full unigene identifiers, individual-sample FPKM values, group means, plotted values, and the original DESeq2 log2FC and FDR values are provided in Table S4. The 15 genes selected for RT-qPCR are marked with asterisks.

In the transport-related panel, the annotated MFS transporter DN6019* and amino acid transport and metabolism protein DN7069* were downregulated at both L-Arg concentrations relative to CK. The InterPro-annotated putative transporter DN2957* was upregulated at 2.5 mM but downregulated at 30 mM. In contrast, the phosphate permease candidate DN10881* was upregulated at both concentrations, whereas the integral membrane protein candidate DN2618* was downregulated at 2.5 mM and upregulated at 30 mM. These genes therefore did not show uniform transcriptional repression (Table S4).

In the secondary metabolism panel, the phenol 2-monooxygenase candidate DN15761* was downregulated at both concentrations relative to CK, although its transcript level increased at 30 mM relative to 2.5 mM. The pyruvate oxidoreductase candidate DN4252* was upregulated in all three comparisons. Other candidates, including DN14447, DN22573, DN8089, and DN1569, showed lower transcript levels under L-Arg treatment (Table S4).

In the degradative enzyme panel, the chitin deacetylase candidate DN29494* and secreted protein candidate DN1795* were downregulated at both concentrations relative to CK. The pectate lyase candidate DN4898* was downregulated at 2.5 mM but upregulated at 30 mM, whereas the dipeptidyl-peptidase candidate DN282* was upregulated in all three comparisons. Lower transcript levels were also observed for several other annotated peptidases and lipases, including DN741, DN3841, DN4145, and DN2086 (Table S4). These annotations cover distinct enzyme and protein classes and should not all be interpreted as confirmed CWDEs.

The organic substance metabolism panel also showed mixed responses. DN1711* was downregulated at 2.5 mM and upregulated at 30 mM relative to CK, whereas DN1864* was downregulated at both concentrations. DN1741* and DN20386* were upregulated relative to CK but had lower transcript levels at 30 mM than at 2.5 mM (Table S4).

#### 3.3.5. The RT-qPCR Validation Experiment

To compare RNA-seq expression patterns with an independent assay, 15 representative DEGs from the four functional categories shown in Figure 6 (marked with asterisks) were selected for RT-qPCR analysis. As shown in Figure 7, the RT-qPCR profiles generally followed the RNA-seq FPKM trends across the 0, 2.5, and 30 mM L-Arg treatments. This agreement supports the expression trends of these selected genes.

## 4. Discussion

In the preliminary screen, all nine amino acids were tested at 30 mM. Ala, Gly, Glu, Asp, Lys, and Arg reduced mycelial growth to varying degrees, whereas Leu, Val, and GABA promoted growth under the tested culture conditions (Figure S1). L-Arg was selected for further concentration–response experiments because it showed the strongest in vitro inhibition in this screen. In the single fruit assay, more decay was observed in the 30 mM group than in the 2.5–20 mM groups. The screening therefore identified an in vitro inhibitory treatment, but did not establish a suitable concentration for fruit protection. The different responses in culture and in fruit underline the need to evaluate antifungal activity and host responses separately.

The genus *Diaporthe* includes plant pathogens, some of which cause postharvest fruit rot, as well as species that produce bioactive metabolites [47,48,49]. In vitro, mycelial growth inhibition increased with L-Arg concentration, whereas the single fruit assay showed a different pattern. Groups receiving 2.5–20 mM L-Arg had lower observed disease indices than the water control, while the 30 mM group had a disease index close to the control. Because no uninoculated 30 mM L-Arg fruit group was included, an effect of the treatment on the fruit itself cannot be excluded. The 30 mM soaking solution was alkaline (pH 10.55, compared with 5.55 for the water control). Its alkalinity may have contributed to the higher disease index at this dose. Osmotic or nutrient-related effects of L-Arg also remain possible; pH-matched and uninoculated-fruit controls are needed to distinguish these explanations. In another study, GABA inhibited growth of a postharvest fungal pathogen in vitro [50], whereas it promoted growth in our preliminary screen.

Functional studies in other fungi have linked the ABC transporters BcatrB in *Botrytis cinerea* and Abc3 in *Magnaporthe grisea* to host-associated stress tolerance and virulence, while the MFS transporter Bcmfs1 protects *B. cinerea* against natural toxic compounds and fungicides [51,52,53]. Fungal ABC efflux pumps also influence sensitivity to antifungal compounds [54,55]. In the present study, however, transport-related candidates showed mixed transcriptional responses: DN6019* and DN7069* were downregulated at both concentrations, the putative transporter DN2957* and DN2618* changed direction between treatments, and DN10881* was upregulated at both concentrations (Figure 6; Table S4). These data identify candidates for further study but do not demonstrate reduced membrane transport activity, ATP availability, or virulence in *D. nobilis*.

Fungal secondary metabolism produces diverse compounds involved in ecological interactions, defense, and development [56,57]. In this study, several annotated metabolic candidates, including DN15761*, DN14447, DN22573, and DN8089, had lower transcript levels under L-Arg treatment, whereas DN4252* was upregulated (Figure 6; Table S4). Comparative genomic and infection-transcriptomic analyses in *Diaporthe* have identified secondary-metabolism gene clusters and infection-associated expression changes [58]. Such evidence provides a basis for selecting candidates, but does not establish that the genes examined here determine virulence. Neither toxin production nor secondary-metabolite concentrations were measured in this study.

Fungal plant cell wall-degrading enzymes can contribute to tissue breakdown and disease development; for example, deletion of Bcpg1 reduces the virulence of *B. cinerea* [59,60]. Biochemical and genetic studies of *B. cinerea* polygalacturonases, together with comparative fungal secretome analyses, further support roles for particular enzymes in degradation and pathogenesis [61,62]. In our data, DN29494* and DN1795* were downregulated at both L-Arg concentrations, but the pectate lyase candidate DN4898* was upregulated at 30 mM and the peptidase candidate DN282* was upregulated at both concentrations (Figure 6; Table S4). The response therefore cannot be described as uniform suppression of CWDEs or degradative enzymes. These results concern transcript levels in PDA-grown mycelia; enzyme activity, secretion, and contribution to virulence during host infection remain untested.

The KEGG results indicate that amino acid metabolism is a recurring feature of the response to L-Arg, particularly glycine, serine and threonine metabolism and arginine and proline metabolism. Expression changes in ribosomal proteins (DN1711*), kinesin-like protein A (DN1864*), tRNA-splicing effector (DN1741*), opsin-1 (DN20386*), and DN4623 also suggest changes in cellular functions under high L-Arg exposure (Figure 6 and Figure 7). Nutrient-dependent repression of arginine metabolism has been described in *Streptococcus faecalis* [63] and *Bacillus subtilis* [64], providing a possible context for these observations. However, the present data do not demonstrate the same regulatory mechanism in *D. nobilis*. The 30 mM treatment produced both upregulated and downregulated DEGs, and neither enrichment analysis nor the number of downregulated genes alone establishes a global metabolic shutdown. Measurements of enzyme activity and metabolite flux would be needed to test this interpretation.

The assembly also limits the interpretation of gene counts and enrichment ratios. Selecting one representative per Trinity gene group reduces within-group transcript multiplicity, but does not establish one-to-one correspondence with genomic loci. The absence of exact duplicate sequences does not exclude near-identical sequences, allelic variants, or fragments assigned to different groups. Enrichment ratios therefore describe the assembled and annotated unigene set, and may be affected by residual redundancy and the choice of background. The 1.1% duplicated BUSCO figure applies to conserved ortholog groups and does not quantify redundancy across all 44,050 unigenes.

Several limitations should be acknowledged. First, the fruit assay was conducted once, and the pooled incidence and disease-index values were not statistically tested. The higher observed decay at 30 mM cannot be attributed solely to fungal responses. The soaking-solution pH increased markedly with L-Arg concentration, whereas the pH of the final solidified PDA was not measured. Without pH-matched or uninoculated-fruit controls, a contribution of alkalinity, osmolality, or host injury to the 30 mM rebound remains unresolved. Second, transcriptomic profiling was restricted to in vitro PDA-grown mycelia and omitted the concentration with the lowest observed fruit disease index (20 mM), leaving in planta infection stage dynamics uncharacterized. The dispersion of the 2.5 mM RNA-seq replicates, particularly A2.5-1, further limits confidence in low-dose-specific expression patterns. Third, the proposed mechanisms rely predominantly on transcriptomic profiling without direct functional (e.g., gene knockouts) or biochemical (e.g., CWDE activity, mycotoxin quantification) validation. The species-level assignment of BLBF-1 remains provisional because additional loci such as TEF1 and TUB2 were not sequenced. Finally, evaluations were performed using a single fungal strain under controlled laboratory conditions, lacking multi-isolate comparisons and commercial storage validation. Future research should incorporate uninoculated fruit physiological controls, in planta dual RNA-seq across key infection stages (including 20 mM), functional knockout and enzymatic assays, and pilot-scale commercial storage trials using diverse strains to validate the practical efficacy and molecular mechanisms of L-Arg.

## 5. Conclusions

In vitro, L-Arg inhibited the mycelial growth of *D. nobilis* more strongly at higher concentrations. In the single fruit assay, the 2.5–20 mM groups had lower observed disease incidence and disease indices than the water control, whereas the 30 mM group had values close to the control. These fruit observations were pooled from one experiment and were not statistically tested, so they do not establish protective efficacy. Independent fruit assays with uninoculated controls and pH- and osmolality-matched treatments, together with measurements during infection, are needed to investigate the difference between the culture and fruit results.

## Figures and Tables

**Figure 1:**
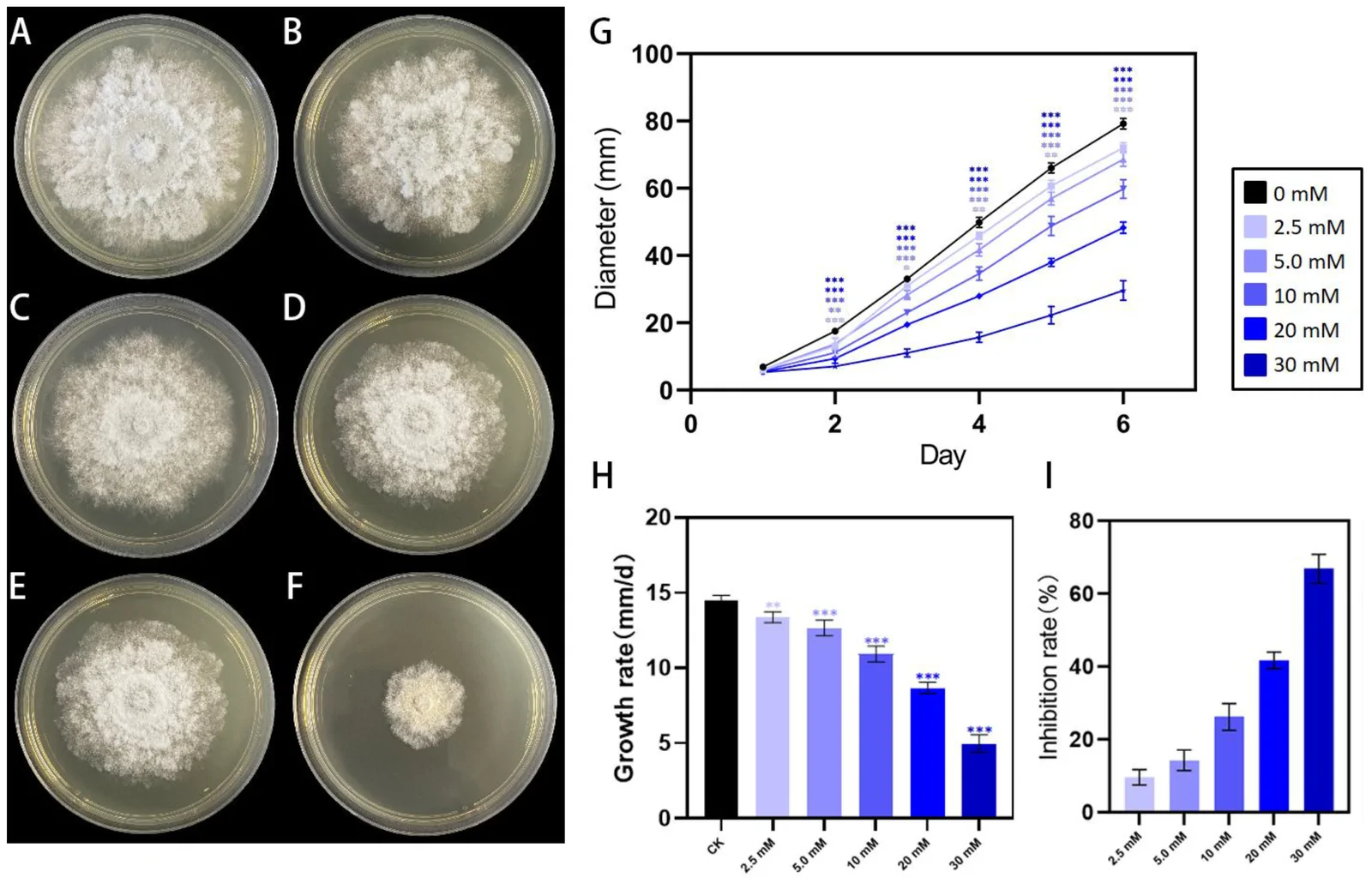
Inhibitory effect of L-Arg on the mycelial growth of *D. nobilis*. (**A**–**F**) Colony morphology of *D. nobilis* cultured on PDA plates supplemented with different concentrations of L-Arg: (**A**) 0 mM (CK), (**B**) 2.5 mM, (**C**) 5.0 mM, (**D**) 10 mM, (**E**) 20 mM, and (**F**) 30 mM. (**G**) Cumulative colony diameter extension (mm) from Day 1 to Day 6 (*n* = 5 independent biological replicates per treatment). (**H**) Mean daily mycelial growth rate (mm/d) across consecutive 24 h intervals (Days 1–6, *n* = 5). (**I**) Inhibition rate (%) of mycelial growth at different concentrations of L-Arg on Day 6 (*n* = 5). Data are presented as mean ± SD. Asterisks indicate significant differences compared with the CK according to one-way ANOVA followed by Dunnett’s post hoc test (* *p* < 0.05, ** *p* < 0.01, *** *p* < 0.001).

**Figure 2:**
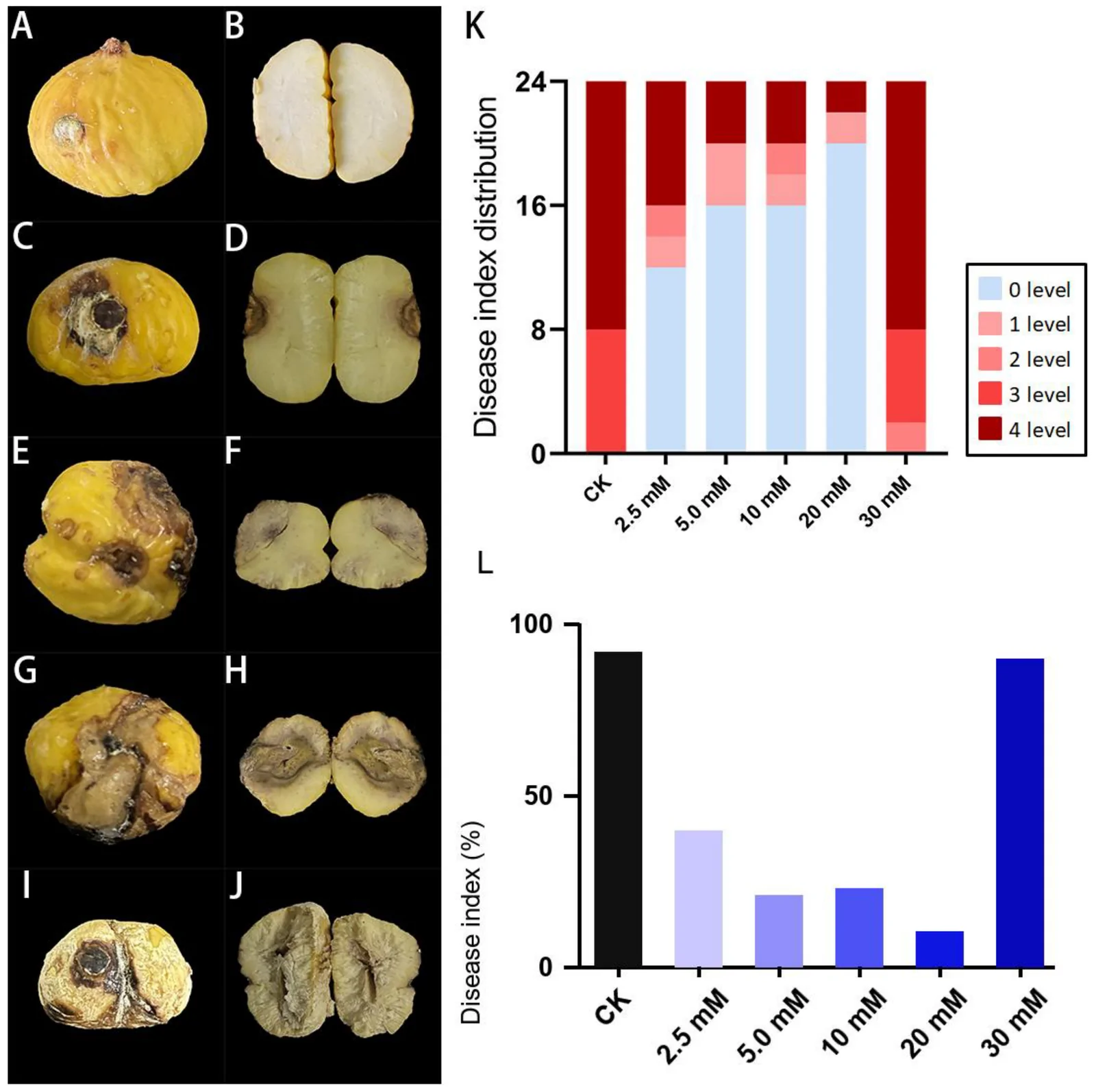
Effect of L-Arg concentration on chestnut decay after inoculation with *D. nobilis*. (**A**–**J**) Representative external and internal symptoms by disease grade: (**A**,**B**) grade 0, (**C**,**D**) grade 1, (**E**,**F**) grade 2, (**G**,**H**) grade 3, and (**I**,**J**) grade 4. (**K**) Counts of fruits in each disease grade (0–4) by treatment. (**L**) Disease index calculated from the grade counts. Each treatment comprised 24 fruits. Disease indices are pooled descriptive values; no statistical test was applied to the fruit data.

**Figure 3:**
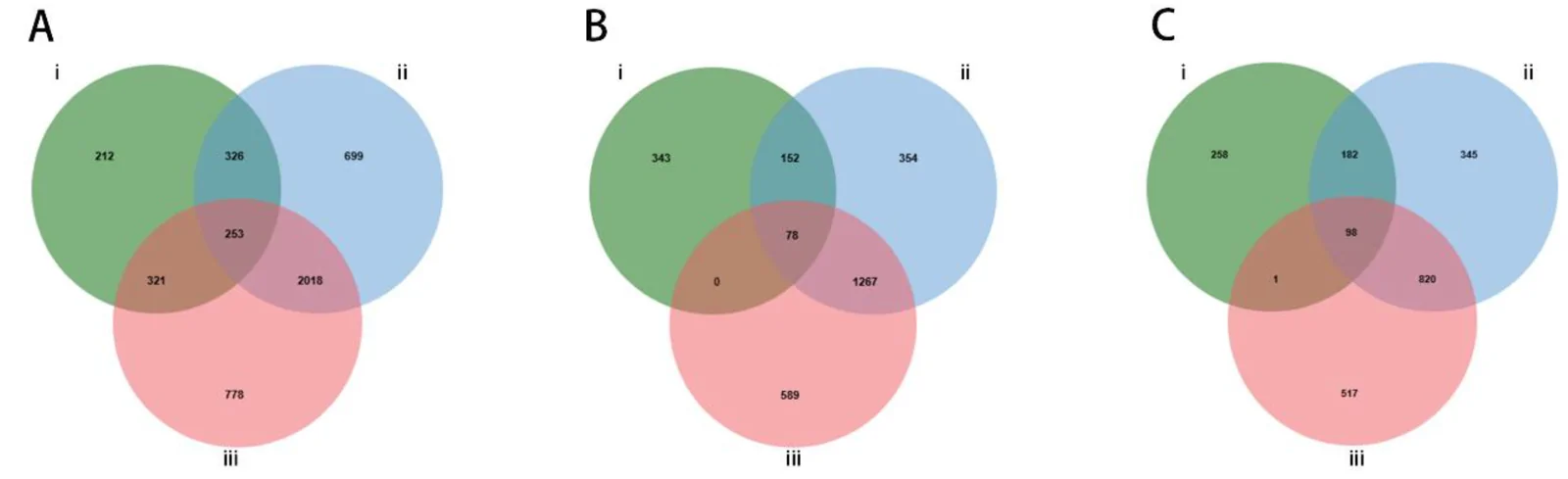
Venn diagrams of differentially expressed genes (DEGs) in *D. nobilis* under different L-Arg treatments. (**A**) Venn diagram showing the overlap of all identified DEGs (|log2 (Fold Change)| ≥ 1 and FDR < 0.01) among the three comparison groups. (**B**) Venn diagram of significantly upregulated DEGs. (**C**) Venn diagram of significantly downregulated DEGs. Roman numerals and colors represent the three comparison groups: green (i), CK vs. 2.5 mM L-Arg; blue (ii), CK vs. 30 mM L-Arg; red (iii), 2.5 mM L-Arg vs. 30 mM L-Arg. Overlapping regions indicate common DEGs shared between or among the respective comparison groups.

**Figure 4:**
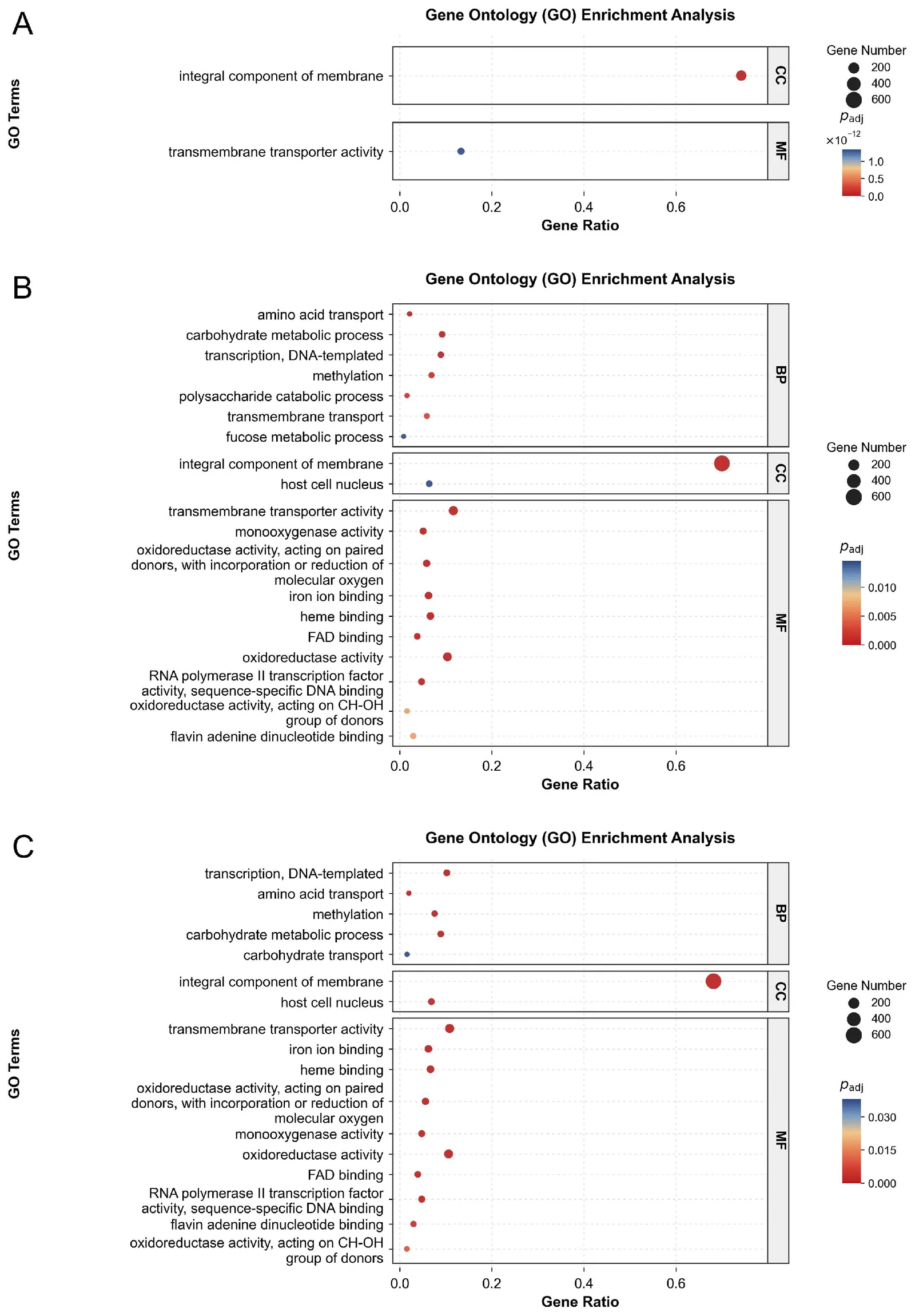
Gene Ontology (GO) enrichment of differentially expressed unigenes in *D. nobilis* under L-Arg treatments: (**A**) 2.5 mM versus CK, (**B**) 30 mM versus CK, and (**C**) 30 mM versus 2.5 mM. Up to 10 terms with BH-adjusted *p* < 0.05 are shown per ontology, ordered from the smallest adjusted *p*-value at the top. BP, biological process; CC, cellular component; MF, molecular function. No BP terms passed the threshold in panel (**A**). Gene Ratio is *k*/*n* as defined in Section 2.4. Bubble size indicates DEG count, and color indicates adjusted *p*-value. Each panel has its own color scale. Complete significance counts, including terms not displayed, are reported in Section 3.3.2.

**Figure 5:**
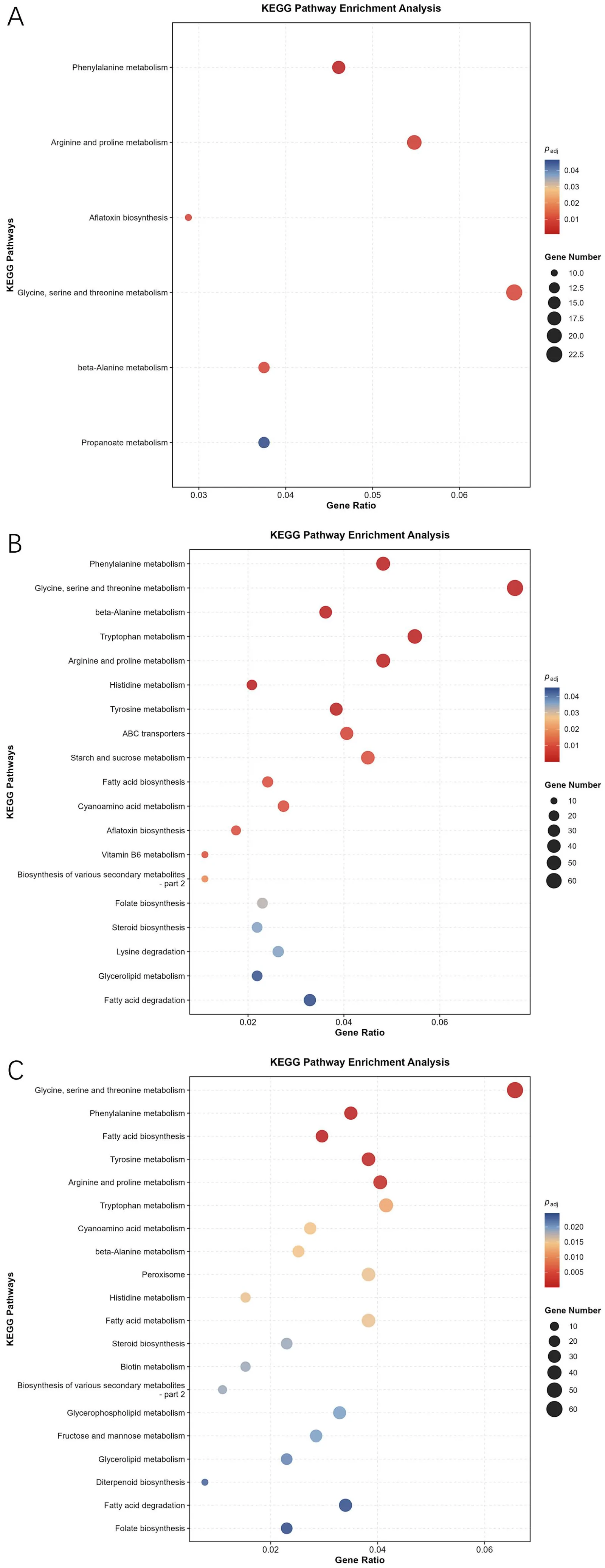
KEGG pathway enrichment of differentially expressed unigenes in *D. nobilis*: (**A**) 2.5 mM L-Arg versus CK, (**B**) 30 mM L-Arg versus CK, and (**C**) 30 mM versus 2.5 mM L-Arg. Up to 20 pathways with BH-adjusted *p* < 0.05 are displayed per comparison, ordered from the smallest adjusted *p*-value at the top. Panels (**A**–**C**) display 6, 19, and 20 pathways, respectively. Gene Ratio is the number of DEGs assigned to a pathway divided by the number represented in the KEGG analysis for that comparison (Section 2.4). Bubble size indicates DEG count; color indicates adjusted *p*-value on the scale shown for each panel. Both upregulated and downregulated DEGs were included.

**Figure 6:**
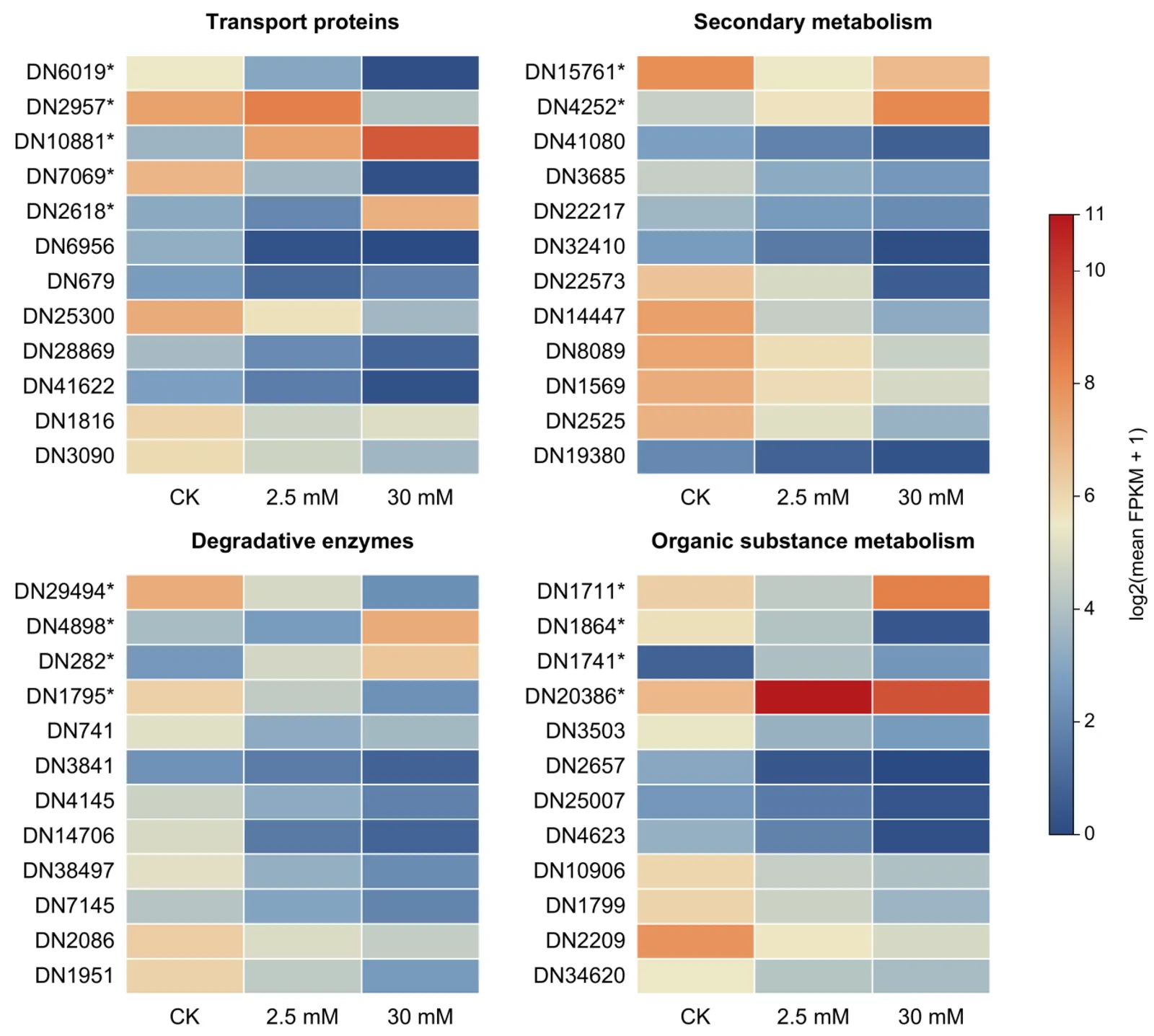
Expression profiles of 48 selected DEGs in *D. nobilis* under CK, 2.5 mM L-Arg, and 30 mM L-Arg treatments. Colors represent log_2_(mean FPKM + 1), where the mean is calculated from three biological replicates per treatment. All four panels use a shared scale from 0 (blue) to 11 (red), without row-wise scaling. Panel headings summarize putative functional annotations. Asterisks mark the 15 genes selected for RT-qPCR analysis in Figure 7. Table S4 provides the full unigene identifiers, FPKM values for all nine samples, group means, plotted values, and log2FC and FDR values for all three comparisons. Blank statistical values in Table S4 indicate that the unigene was not reported in the corresponding original DESeq2 output.

**Figure 7:**
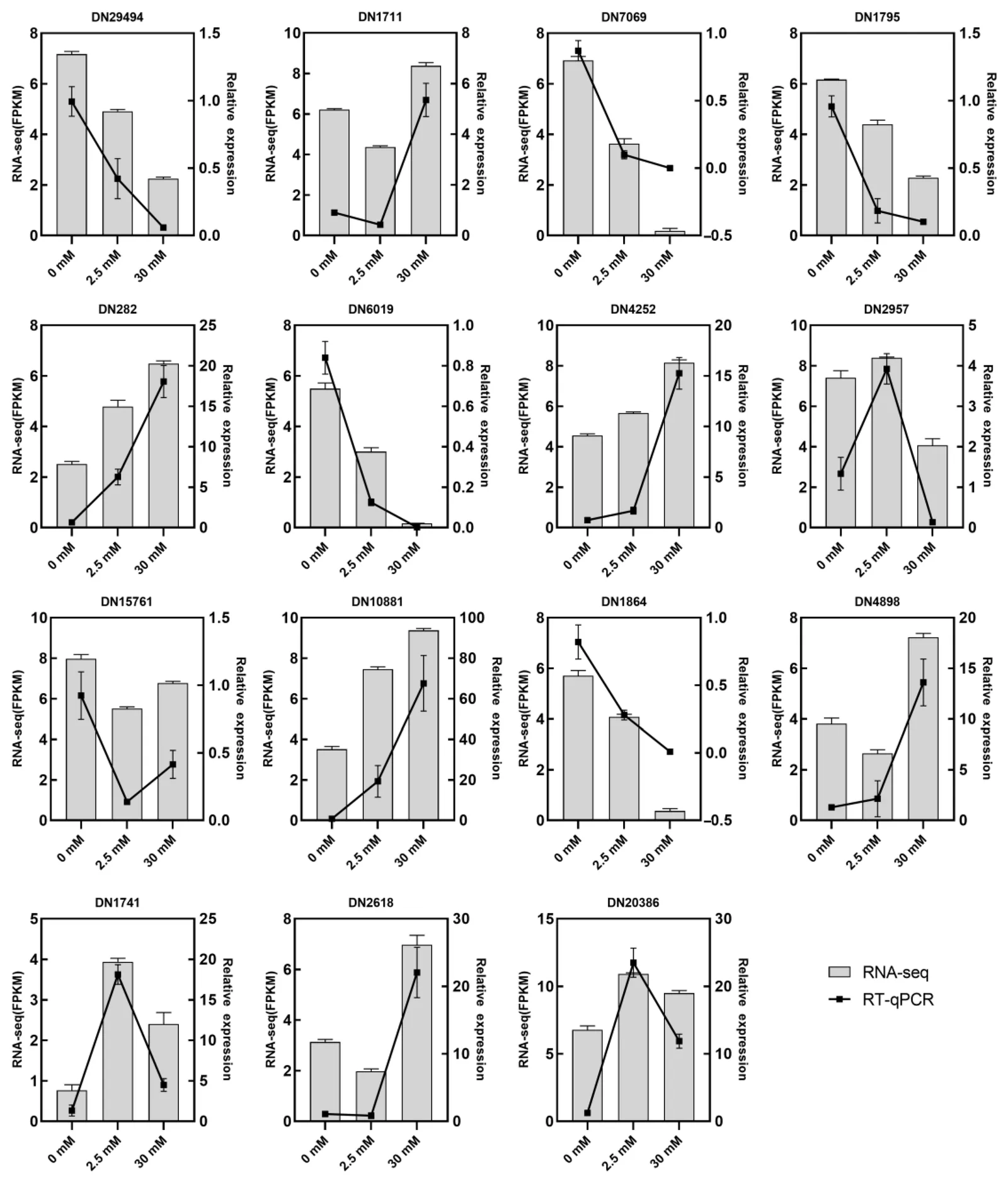
RT-qPCR validation of representative differentially expressed genes (DEGs) in *D. nobilis* under different L-Arg treatments. Fifteen representative DEGs from the four functional categories shown in Figure 6 were analyzed under 0 mM (CK), 2.5 mM, and 30 mM L-Arg treatments to compare their expression trends with the RNA-seq results. Gray bars represent transcript abundance measured by RNA-seq (FPKM values, left *y*-axis), and black lines with square markers indicate relative gene expression levels determined by RT-qPCR (2^−ΔΔCt^ method, right *y*-axis). Data are presented as mean ± SD (*n* = 3). The two assays showed similar expression trends for these selected genes.

## Data Availability

The raw transcriptomic sequencing data generated in this study have been deposited in the NCBI Sequence Read Archive (SRA) database under BioProject accession number PRJNA1228502. The datasets are publicly accessible at PRJNA1228502 Details|Manage Data|Submission Portal.

## Supplementary Materials

The following supporting information can be downloaded at https://www.mdpi.com/article/10.3390/jof12100783/s1. Supplementary Figure S1. Colony diameter of *D. nobilis* on PDA with amino acids (30 mM) over six days. (A) Growth-inhibiting amino acids (Ala, Gly, Glu, Asp, Lys, and Arg); (B) growth-promoting amino acids (Leu, Val, and GABA). CK, unsupplemented PDA. Values are mean ± SD (n = 3). Asterisks show differences from CK at each time point (* *p* < 0.05, ** *p* < 0.01, *** *p* < 0.001; ns, not significant). Figure S2. Heatmap of squared Spearman rank-correlation coefficients (ρ^2^) for pairwise unigene expression profiles among nine RNA-seq libraries: CK1–CK3, A2.5-1–A2.5-3, and A30-1–A30-3 (n = 3 per treatment). The values were calculated for unigenes with FPKM > 0.1 in both samples of each pair. The 2.5 mM replicates show greater variation; some between-treatment values exceed a within-group value. Labels Arg2.5-1–Arg2.5-3 and Arg30-1–Arg30-3 in the plot correspond to A2.5-1–A2.5-3 and A30-1–A30-3 in the manuscript. Figure S3. Principal component analysis (PCA) of the nine RNA-seq libraries. CK1–CK3, A25-1–A25-3, and A30-1–A30-3 denote three biological replicates per treatment. The 2.5 mM samples show greater dispersion than the CK and 30 mM groups; A25-1 (called A2.5-1 in the manuscript) is especially separated from the other low-dose samples. CK3 is close to the other CK samples in this plot. The figure is descriptive and does not exclude sample effects in differential-expression analysis. Supplementary Table S1. Target genes and the respective PCR primers. Supplementary Table S1 (continued). Amplification-curve metrics for the RT-qPCR assays. Supplementary Table S2. Base information statistics. Supplementary Table S3. Differentially Expressed Genes (DEGs) Count Table. Supplementary Table S4. Source data for Figure 6. The accompanying file Supplementary Tables S4 and S5 provides all 48 plotted unigenes, their full Trinity identifiers, individual-sample FPKM values for the nine RNA-seq libraries, arithmetic group means, and the plotted log_2_;(mean FPKM + 1) values. The original DESeq2 log2FC, FDR, and direction classifications are included for all three comparisons. Positive log2FC indicates higher expression in the first named treatment: 2.5 mM versus CK, 30 mM versus CK, or 30 mM versus 2.5 mM. Blank log2FC or FDR cells indicate absence from the original comparison file; no missing values were imputed. Supplementary Table S5. Direction patterns of the 253 common DEGs. The 78 up/up/up and 98 down/down/down DEGs have consistent directions across all three comparisons. The other 77 have mixed directions. The full gene list and the original log2FC and FDR values are supplied in the Table S5 worksheet of the accompanying Excel file. All 253 genes met the original DEG criteria in each comparison (FDR < 0.01 and |log2FC| ≥ 1). Supplementary Table S6. BUSCO assessment of the final unigene assembly. BUSCO v5.8.0 was run in transcriptome mode on 44,050 representative unigene sequences using the sordariomycetes_odb10 dataset (3817 ortholog groups). Percentages are calculated against the total number of ortholog groups.
