# Supplementary material for: Inhibitory Effects and Transcriptomic Insights of L-Arginine Against Diaporthe nobilis
Source: J Fungi (Basel). 2026 Oct 19;12(10):783. doi: 10.3390/jof12100783 (PMC13706520; doi:10.3390/jof12100783)
Supplement: Supplementary file 1 [file jof-12-00783-s001.zip › Supplementary materials.pdf]

Supplementary material

Supplementary Table S1. Target genes and the respective PCR primers

Gene		5'--3'	Putative Gene Name	Description
actin	F	CATGAAGTGTGATGTCGATGTCC	Actin	Internal reference gene
	R	CAGACGGAGTACTTTCGCTCG		
DN29494	F	GCTGCACTGCCGTATCATTG	CDA1 / Chitin deacetylase	Chitin deacetylase 1
	R	CTGCTGCTTGAACGCTTCG		
DN1711	F	TCTCCTCGTCAACGCCATC	Ribosomal protein	Ribosomal protein L7Ae/L30e/S12e family
	R	CCAGCAAGCAAGGTAGTTATGT		
DN7069	F	TCGACTGGTTCAAGGATAGAGG	Uncharacterized protein	Amino acid transport and metabolism protein
	R	GAGGCAGGAGAGTAGCACATAT		
DN1795	F	GATGTCGGCAATGGCAAGG	*BAS2* / Secreted protein	Biotrophy-associated secreted protein 2
	R	GATGGTAGCAGCAGCGTTC		
DN282	F	TTGTTGGTGGTTGCCTATGTG	*DPP4* / Dipeptidyl peptidase	Extracellular dipeptidyl-peptidase 4
	R	ACTGAGGTTGAGGAGCGATG		
DN6019	F	CGTCACCAGCAAGTACAAGAA	MFS transporter	Major facilitator superfamily transporter
	R	GCGAGGAAGAGCGAGATCA		
DN4252	F	TTGCCATCGCCACCACAT	Pyruvate oxidoreductase	Pyruvate ferredoxin oxidoreductase beta subunit
	R	GAAGACGCCCAGAAAGTAGAAG		
DN2957	F	AACCACTCTCAACCACCTCTC	putative transporter	Putative transporter
	R	TGGCCTTGGAGAAGTACATCTT		
DN15761	F	GTGACGCAGGACAAGAGAAC	Phenol 2-monooxygenase	FAD-dependent phenol 2-monooxygenase
	R	GGATGATGAGGATAGAGCCGAA		
DN10881	F	CTTCGCCGCCATCATATTCC	*pho-4* / Phosphate permease	Phosphate-repressible phosphate permease
	R	CCTTCCAGACGATCAGCATTG		
DN1864	F	TACGAGCAAGGACTGGAGATTA	*klpA* / Kinesin motor	Kinesin-like protein A
	R	CGAGATGGATCACGGAGACT		
DN4898	F	CACTCTCGTCTCCAGCCTTAT	*plyA* / Pectate lyase	Pectate lyase A
	R	GCAGCGGTCTTGAACTCCT		
DN1741	F	GGCAGGAGCAGTACCACAT	tRNA-splicing effector	tRNA-splicing endonuclease positive effector
	R	GCGTGACCACTTATCATCTTCT		
DN2618	F	AACCAGACCGACATCACTCAT	Transmembrane protein	Uncharacterized integral membrane protein
	R	CTTAGGACCTCCACCAATCGT		
DN20386	F	CTGGCTCTGTTCACATTCCTTC	*nop-1* / Opsin-1	Opsin-1 / Rhodopsin-like membrane protein
	R	AATGCTGCGGTGGCTGAT		

Supplementary Table S1 (continued). Amplification-curve metrics for the RT-qPCR assays

Target (assay)	Efficiency (%) mean ± SD	Curve-fit R^2 mean ± SD	n
actin	86.6 ± 5.9	0.9991 ± 0.0013	169
DN29494	89.6 ± 3.7	0.9996 ± 0.0004	27
DN1711	91.0 ± 6.8	0.9992 ± 0.0008	27
DN7069	89.9 ± 3.4	0.9995 ± 0.0008	27
DN1795	89.6 ± 4.7	0.9994 ± 0.0008	27
DN282	100.6 ± 13.6	0.9898 ± 0.0183	20
DN6019	89.7 ± 3.7	0.9995 ± 0.0006	25
DN4252	90.3 ± 11.5	0.9980 ± 0.0027	25
DN2957	90.2 ± 4.6	0.9991 ± 0.0010	27
DN15761	86.8 ± 5.7	0.9982 ± 0.0037	26
DN10881	89.0 ± 6.7	0.9985 ± 0.0013	23
DN1864	87.4 ± 5.1	0.9988 ± 0.0013	26
DN4898	88.8 ± 3.5	0.9994 ± 0.0004	27
DN1741	90.2 ± 4.2	0.9995 ± 0.0006	27
DN2618	88.3 ± 4.8	0.9993 ± 0.0005	27
DN20386	86.7 ± 2.8	0.9995 ± 0.0012	27

Supplementary Table S2. Base information statistics

**Sample**	**CleanData(bp)**	**GC(%)**	**Q30(%)**
Arg2.5-1	10,583,680,200	54.84%	94.39%
Arg2.5-2	10,376,371,200	55.67%	94.36%
Arg2.5-3	10,050,920,100	55.68%	94.33%
Arg30-1	9,270,846,900	55.20%	94.47%
Arg30-2	10,137,659,700	55.67%	94.55%
Arg30-3	9,755,761,500	55.44%	94.61%
CK-1	9,733,505,700	55.79%	94.43%
CK-2	10,203,585,300	55.88%	94.54%
CK-3	9,994,708,200	55.58%	94.44%

Supplementary Table S3. Differentially Expressed Genes (DEGs) Count Table

**Group**	**All DEG**	**Up**	**Down**
CK vs 2.5 mM	1,112	573	539
CK vs 30 mM	3,296	1,851	1,445
2.5 mM vs 30 mM	3,370	1,934	1,436

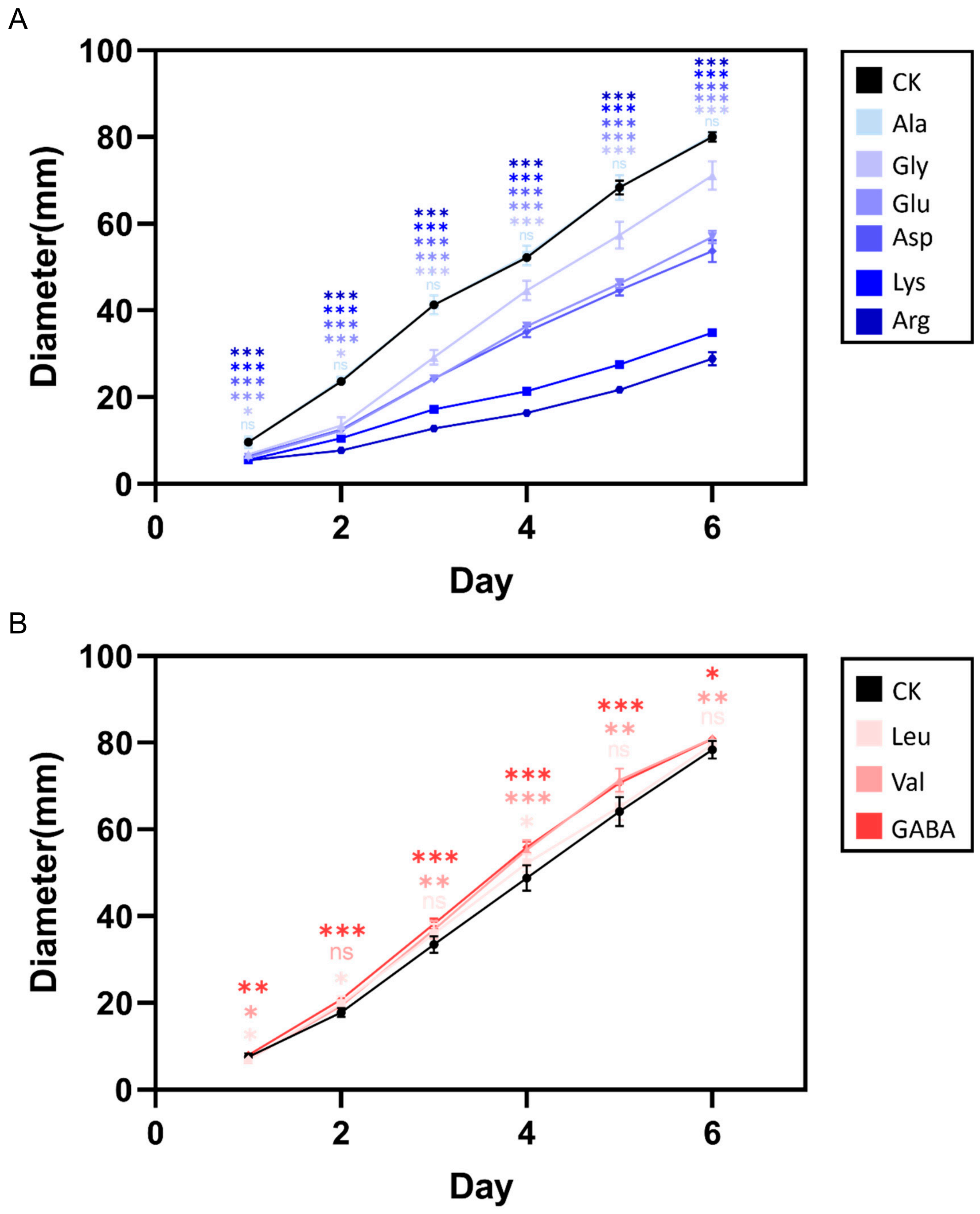

Supplementary Figure S1. Colony diameter of *D. nobilis* on PDA with amino acids (30 mM) over six days. (A) Growth-inhibiting amino acids (Ala, Gly, Glu, Asp, Lys, and Arg); (B) growth-promoting amino acids (Leu, Val, and GABA). CK, unsupplemented PDA. Values are mean ± SD (n = 3). Asterisks show differences from CK at each time point (*p < 0.05, **p < 0.01, ***p < 0.001; ns, not significant).

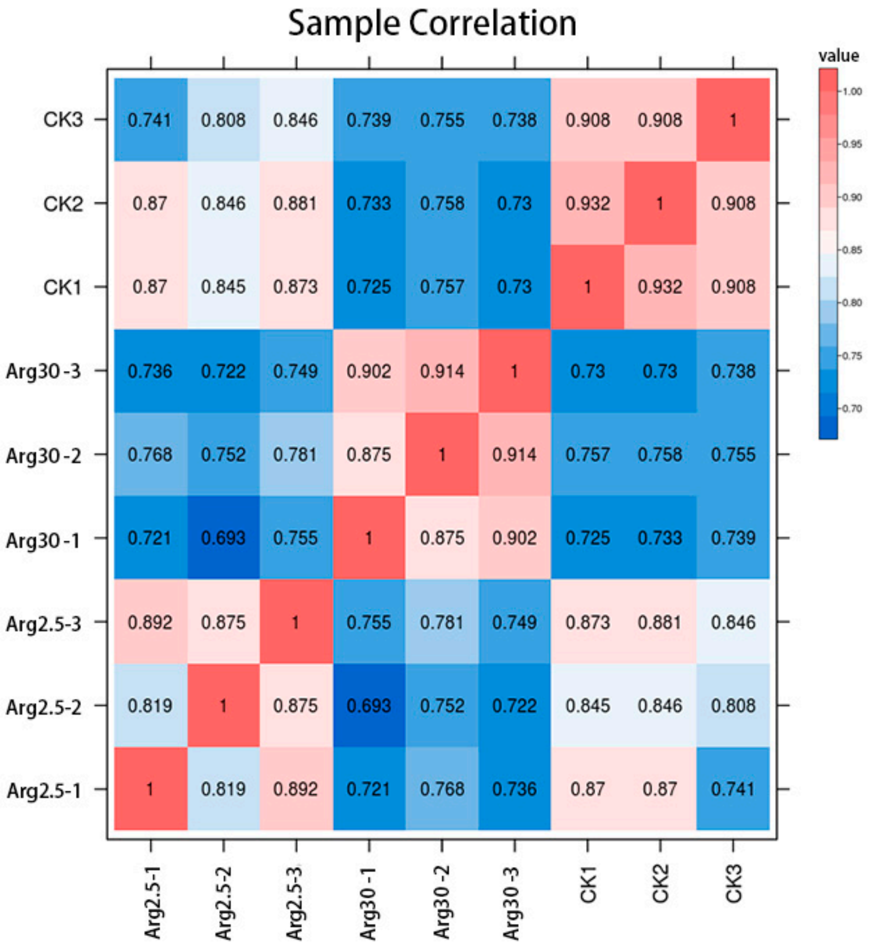

Figure S2. Heatmap of squared Spearman rank-correlation coefficients (ρ^2) for pairwise unigene expression profiles among nine RNA-seq libraries: CK1–CK3, A2.5-1–A2.5-3, and A30-1–A30-3 (n = 3 per treatment). The values were calculated for unigenes with FPKM > 0.1 in both samples of each pair. The 2.5 mM replicates show greater variation; some between-treatment values exceed a within-group value. Labels Arg2.5-1–Arg2.5-3 and Arg30-1–Arg30-3 in the plot correspond to A2.5-1–A2.5-3 and A30-1–A30-3 in the manuscript.

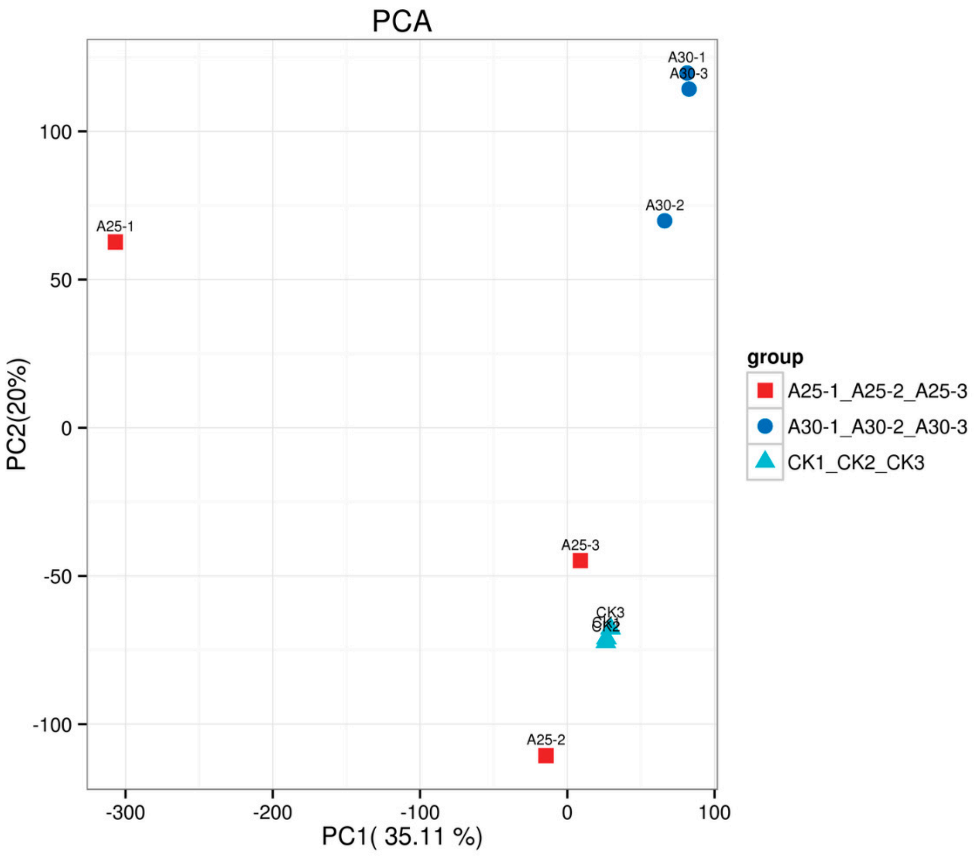

Figure S3. Principal component analysis (PCA) of the nine RNA-seq libraries. CK1–CK3, A25-1–A25-3, and A30-1–A30-3 denote three biological replicates per treatment. The 2.5 mM samples show greater dispersion than the CK and 30 mM groups; A25-1 (called A2.5-1 in the manuscript) is especially separated from the other low-dose samples. CK3 is close to the other CK samples in this plot. The figure is descriptive and does not exclude sample effects in differential-expression analysis.

Supplementary Table S4. Source data for Figure 6.

The accompanying file Supplementary Tables S4-S5.xlsx provides all 48 plotted unigenes, their full Trinity identifiers, individual-sample FPKM values for the nine RNA-seq libraries, arithmetic group means, and the plotted $\log_2$(mean FPKM + 1) values. The original DESeq2 log2FC, FDR, and direction classifications are included for all three comparisons. Positive log2FC indicates higher expression in the first named treatment: 2.5 mM versus CK, 30 mM versus CK, or 30 mM versus 2.5 mM. Blank log2FC or FDR cells indicate absence from the original comparison file; no missing values were imputed.

Supplementary Table S5. Direction patterns of the 253 common DEGs.

2.5 mM versus CK	30 mM versus CK	30 mM versus 2.5 mM	Number of DEGs
down	down	down	98
up	up	up	78
up	down	down	35
down	up	up	34
up	up	down	7
down	down	up	1

The 78 up/up/up and 98 down/down/down DEGs have consistent directions across all three comparisons. The other 77 have mixed directions. The full gene list and the original log2FC and FDR values are supplied in the Table S5 worksheet of the accompanying Excel file. All 253 genes met the original DEG criteria in each comparison (FDR < 0.01 and $|\log2FC| \geqslant 1$).

Supplementary Table S6. BUSCO assessment of the final unigene assembly.

BUSCO v5.8.0 was run in transcriptome mode on 44,050 representative unigene sequences using the sordariomycetes_odb10 dataset (3,817 ortholog groups). Percentages are calculated against the total number of ortholog groups.

BUSCO category	Number	Percentage
Complete (C)	3,730	97.7%
Complete and single-copy (S)	3,689	96.6%
Complete and duplicated (D)	41	1.1%
Fragmented (F)	32	0.8%
Missing (M)	55	1.4%
Total BUSCO groups	3,817	100%
